# Retraction: USP22 protects against myocardial ischemia–reperfusion injury via the SIRT1-p53/SLC7A11–dependent inhibition of ferroptosis–induced cardiomyocyte death

**DOI:** 10.3389/fphys.2025.1641580

**Published:** 2025-06-12

**Authors:** Frontiers Editorial Office

The journal retracts the 21 October 2020 article cited above. Following publication, concerns were raised regarding the integrity of the images in the published figures. The authors failed to provide a satisfactory explanation during the investigation, which was conducted in accordance with Frontiers’ policies.

This retraction was approved by the Chief Editors of Frontiers of Physiology and the Chief Executive Editor of Frontiers. The authors received a communication regarding the retraction and had a chance to respond. This communication is recorded by the publisher.

